# Forest fragmentation and heterogeneity shape the occurrence of woodpecker species in Central Europe

**DOI:** 10.1038/s41598-025-04832-5

**Published:** 2025-07-01

**Authors:** Michał Bełcik, Bartłomiej Woźniak, Piotr Skórka

**Affiliations:** 1 Institute of Nature Conservation, Polish Academy of Sciences, Mickiewicza 33, 31-120 Kraków, Poland; 2 Department of Forest Zoology and Wildlife Management, Institute of Forest Sciences, Warsaw University of Life Sciences – SGGW, Nowoursynowska 159, 02-776 Warsaw, Poland

**Keywords:** Woodpeckers, Bird diversity, Connectivity, Habitat quality, Landscape, Woodland, Forest ecology, Zoology

## Abstract

The study investigates the impact of fragmentation metrics and other forest characteristics on the occurrence and richness of woodpecker species in 163 forest patches in Southern Poland. Generalised linear mixed models were used to estimate the influence of fragmentation metrics (patch size, nearest-neighbour distance, proximity index, patch shape) and forest stand features (age, proportion of coniferous tree species, proportion of dominant tree species) on woodpecker presence and woodpecker species richness. Eight woodpecker species were identified during surveys, and the study found that forest patch size positively correlated with the probability of occurrence for the great spotted woodpecker and black woodpecker but negatively with the occurrence of wryneck. The nearest-neighbour distance between two forest patches and the proximity index were negatively correlated with the occurrence of the lesser spotted woodpecker. The shape index negatively influenced the occurrence of the great spotted woodpecker but positively the occurrence of the wryneck. The European green woodpecker occurrence probability decreased with the proportion of coniferous tree species. Woodpecker species richness was positively associated with forest patch size and age, but negatively with the proportions of coniferous trees and dominant tree species. These findings indicate that forest fragmentation is a major driver of woodpecker species occurrence and richness, along with habitat quality characteristics.

**Supplementary Information:**

The online version contains supplementary material available at 10.1038/s41598-025-04832-5.

## Introduction

Habitat loss and fragmentation drive major ecological processes (e.g. dispersal, population viability, and species interactions) across the globe^1–3^. According to the classic concepts of island biogeography^4^ and the metapopulation theory^5,6^, the isolation of habitat patches negatively affects species occurrence and abundance in fragmented landscapes. These concepts suggest that larger, less isolated habitat patches have a greater chance of being occupied by a given species and support higher population densities than smaller, more isolated patches^4,5^.

Woodlands represent a critical habitat for many bird species, including keystone and ecosystem engineers such as woodpeckers^7,8^. Woodlands currently face substantial anthropogenic pressure^9,10^^.^, primarily due to fragmentation^11^. This disruption significantly impacts biodiversity within these forests, as many species rely on large, contiguous habitats for survival^12–14^. The reduction in patch size and increased isolation can lead to a decline in species richness^15,16^ and the disruption of ecological interactions^17^. Moreover, fragmented forests often create edge effects, where altered conditions at the peripheries of forest patches affect the interior environment, further modifying both inter- and intra-specific interactions^9,17^.

However, certain forest characteristics related to habitat quality may mitigate the negative effects of habitat fragmentation on species populations. Forest age, tree species heterogeneity, and tree cover density can significant impact bird biodiversity^9,10^. Older forests typically provide a variety of microhabitats and a greater availability of deadwood, which support a higher diversity of bird species^18,19^. Additionally, tree species diversity increases habitat heterogeneity and complexity, offering more abundant insect prey and better refuges from predators^20^. Therefore, old, mixed forests with diverse tree species and abundant deadwood tend to support higher bird biodiversity^21^.

To evaluate the effects of forest habitat quality and fragmentation on species diversity, it is useful to focus on an indicator group of species that (i) signal the presence of a range of other species, (ii) act as keystone species, and (iii) are sensitive to specific environmental conditions, serving as early indicators of environmental changes. In this context, woodpeckers are particularly valuable^22,23^. Notably, due to their unique life history, woodpeckers create cavities that are essential for secondary cavity-nesters^24^, leading some species to be considered keystone species^23^. Furthermore, woodpeckers are highly susceptible to environmental changes caused by different management practices, making them excellent biotic indicators of forest biodiversity and health^7,25^. This sensitivity is further evidenced by research showing that woodpecker populations can be negatively impacted by forest fragmentation^26,27^, reinforcing their role as strong indicators of the effects of forest fragmentation on avian diversity.

In this study, we investigated the effects of forest fragmentation and habitat quality on the occurrence of individual woodpecker species, as well as species richness, within forest patches located in a rural landscape in Poland. We hypothesised that (i) the probability of woodpecker species occurrence and species richness would increase with forest patch size, and decrease with increasing spatial isolation and shape index; (ii) woodpecker occurrence and species richness would increase with forest age, and decrease with the homogenisation of tree species composition (i.e. the proportion of dominant species and/or percentage of coniferous species). Finally, we assessed (iii) whether woodpeckers species richness could predict the richness of other bird species observed during surveys.

## Materials and methods

### Study sites

**Fig. 1:**
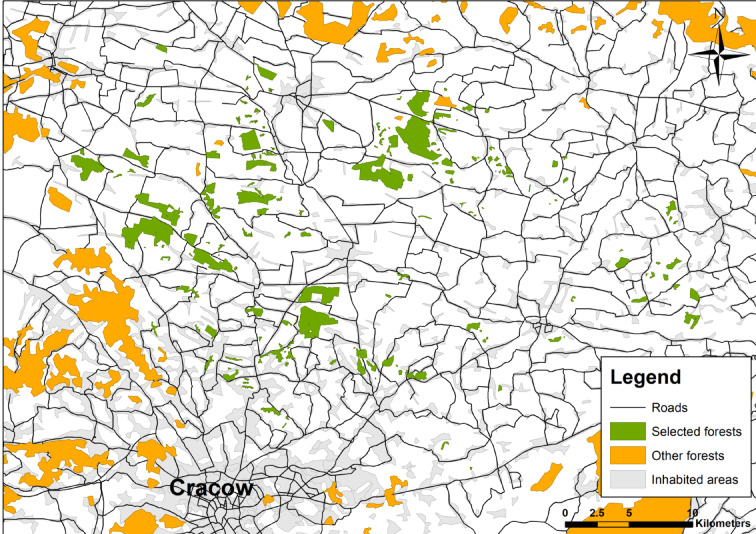
Map of the study area, with study forest patches marked in green and other forests marked in orange. Created by Michał Bełcik using ESRI ArcMap ver. 10.1.

The study area spanned 1097 km^2^ across southern Poland, within the Lesser Poland province, north of Cracow (Fig. 1). Within this area, we selected 163 forest patches distributed in an agricultural landscape, primarily composed of mixed stands managed or supervised by the Polish State Forests Holding. The selected forest patches were isolated from larger, continuous forest complexes and varied in size, degree of isolation, and the amount of surrounding forest patches (Table 1).

**Table 1:** Forest stand parameters and fragmentation metrics of studied forest patches.

Parameter	Type of parameter	Description	Range	Mean ± SD
Forest patch size	Fragmentation variable	Total area of forest patch (in hectares)	0.38–582.33	37.28 ± 89.52
Nearest-neighbour distance	Fragmentation variable	Shortest straight-line distance between a focal patch and its nearest neighbour (in meters)	16.53–3509.19	269.26 ± 701.36
Proximity index	Fragmentation variable	Sum of the sizes of all patches whose edges are within the 2.5 km radius of the focal patch, divided by the square of their distance from the focal patch	0.00–1845.83	78.86 ± 251.92
Shape index	Fragmentation variable	Shape Index of forest stand. Normalized ratio of patch perimeter to area in which the complexity of patch shape is compared to a standard shape (square) of the same size	1.110–3.528	1.790 ± 0.504
Forest age	Stand parameter	Mean age of dominant tree species in main stand storey (in years)	10–112	58.18 ± 24.30
Coniferous species	Stand parameter	Percentage of coniferous species in main stand storey (in %)	0.00–100.00	21.01 ± 26.04
Proportion of dominant species	Stand parameter	The share of dominant tree species in main stand canopy, expressed on an integer scale of 0 to 10 (with 10 representing the highest value),	2–10	-

### Field surveys

Field surveys were conducted between 1 April and 31 May 2017, by a team of three experienced birdwatchers, each with over ten years of experience in performing bird censuses. Each observer was assigned a specific set of forest patches, with each patch visited three times, once during each 20-day rounds (1–20 April, 21 April–10 May, 11–31 May). Surveys commenced at approximately 5 a.m. and typically continued until 11 a.m. During surveys, observers noted the start time and moved through the forest in random directions to cover maximum area. They documented all observed woodpecker species, other bird species, and the exact time when the first individual of each species was heard or seen within a patch^28^. The survey was terminated if no new species were observed for ten minutes^28^.

### Forest characteristics

For each forest patch, we collected a range of parameters that best represent the key characteristics of a forest stand potentially important for woodpecker species (Table 1). These parameters were measured and averaged for each patch. Additionally, we used the Forest Data Bank (www.bdl.lasy.gov.pl) as a data source for some of the patches. When data from the Forest Data Bank was not available, we calculated the necessary parameters according to the guidelines of Forest Bureau for Forest Management and Geodesy^29^. We calculated the forest patch size (in ha) and forest patch isolation for each surveyed forest patch. To measure patch isolation, we used two metrics: the nearest-neighbour distance and the proximity index, which were weakly correlated (r =  − 0.203, *P* = 0.031). All fragmentation metrics were calculated using the Patch Analyst toolbox in ArcGis ver. 10.1^30^. Furthermore, we calculated the shape index of each forest patch, which is the ratio of the patch perimeter to the theoretical perimeter if the patch was a circle. This index measures the compactness of each forest patch (Table 1). In addition to these metrics, we calculated forest stand parameters such as forest age, the percentage of coniferous species, and the proportion of dominant tree species (Table 1).

### Data analysis

We constructed a series of generalised linear mixed models (GLMM) to analyse explanatory variables associated with woodpeckers occurrence and woodpecker species richness. We used “glmmTMB” R package ver. 1.1.10^31^ in R ver. 4.1.2^32^ . To assess whether fragmentation metrics and forest stand characteristics influenced woodpecker species occurrence probability during each survey we used GLMMs with a binomial error distribution and complementary log–log link function (“cloglog”), except in case of models for great spotted woodpecker where “logit” link function was used because model diagnostics indicated better performance. The “cloglog” link function is particularly useful in statistical models when dealing with binary outcomes, in cases where the probability of an event occurring is very small or very large. In such scenarios, the “cloglog” link can provide a better fit than symmetric link functions^33^. We used following explanatory variables: forest patch size, nearest-neighbour distance, proximity index, patch shape index, average tree age in forest patch, percentage share of the dominant tree species, percentage of coniferous species, temperature during surveys, cloud cover and survey duration (in minutes) (Table 1). To account for spatial autocorrelation in GLMMs we used the k-means clustering method^34^ to find spatial clusters in the distribution of forests in our study area. We included these clusters as a random factor. Following variables were log-transformed to reduce the influence of outliers: parch size, nearest-neighbour distance, proximity index, percentage share of the dominant tree species, percentage of coniferous tree species and survey duration. All continuous explanatory variables were also scaled (mean = 0, sd = 1) as this improves model performance and interpretability in general^34^. Forest identity (unique forest number) was included as a random effect. A GLMM with a Conway-Maxwell Poisson error distribution^35^ was used to explain overall woodpecker species richness in forest patches during the surveys. This model was chosen because it was underdispersed and yielded the most satisfactory model diagnostics (Supplementary information). . In this model we used the same explanatory variables as in models for species occurrence.

We also used GLMM with a Conway-Maxwell Poisson error distribution to test the association between the number of woodpeckers species and the richness of other birds during surveys. In these models, forest identity and spatial cluster were included as the random factors.

To validate our GLMMs we used “DHARMa” R package ver. 0.4.7^36^.

To analyse which forest parameters differentiate woodpeckers assemblages and to assess the importance of forest stand parameters, a partial canonical correspondence analysis (partial-CCA) was conducted using the “vegan” R package ver. 2.6–6.1^37^. The explanatory variables included log-transformed fragmentation metrics (nearest-neighbour distance, patch size, proximity index, shape index), forest stand parameters (age of the dominant tree species, percentage of coniferous species, and the proportion of the dominant tree species in the main forest canopy). Geographic coordinates, day of the control and weather conditions during the survey were also assigned as covariates.

## Results

### Observed woodpecker species

Eight woodpecker species were observed across the study area. These included: the great spotted woodpecker (*Dendrocopos major—*found in 138 forest patches), the middle spotted woodpecker (*Leiopicus medius*—found in 7 patches), the lesser spotted woodpecker (*Dryobates minor—*found in 8 patches), the Syrian woodpecker (*Dendrocopos syriacus—*found in 1 patch), the black woodpecker (*Dryocopus martius—*found in 25 patches), the European green woodpecker (*Picus viridis—*found in 24 patches), the grey-headed woodpecker (*Picus canus—*found in 7 patches), and the wryneck (*Jynx torquilla—*found in 19 patches). The mean number of woodpecker species per survey per forest patch was 0.908 (SD ± 0.761).

### Effects of fragmentation metrics on the occurrence of individuals species

**Fig. 2:**
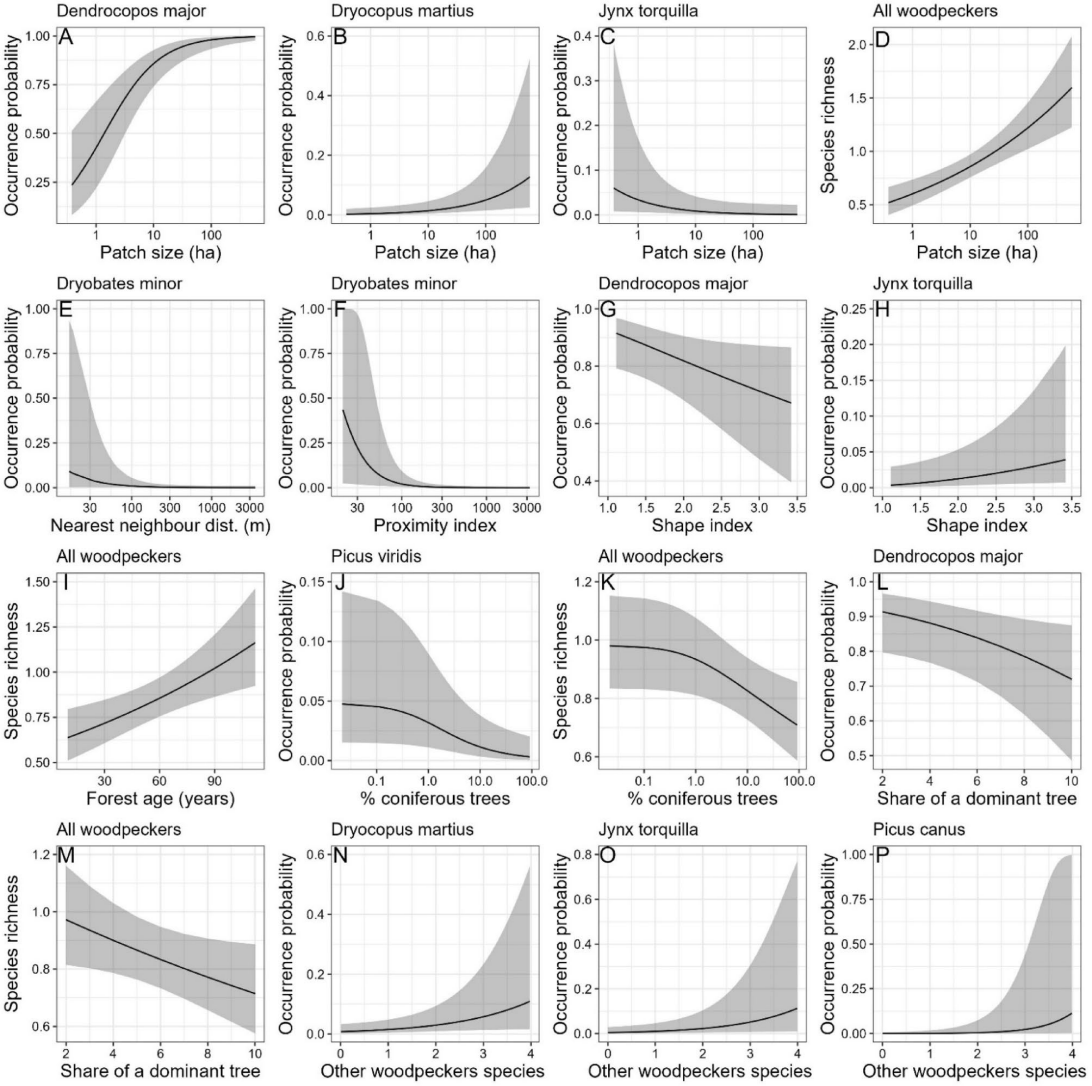
Factors influencing the occurrence probability (**A**–**C**, **E**–**H**, **J**, **L**, **N**–**P**) of woodpeckers and their species richness (**D**, **I**, **K**, **M**). Subplots indicate effects of forest patch size (**A**–**D**), nearest-neighbour distance between two forest patches (**E**), proximity index (the total area of other forest patches around a given patch—**F**), shape index of forest patches (**G**–**H**), forest age (**I**), percentage of coniferous species within the main forest canopy (**J**–**K**), share of dominant tree species within main forest canopy (**L**–**M**), and number of other woodpecker species (**N**–**P**). Results from generalised linear mixed models (see: Table 2).

Habitat fragmentation metrics were significantly associated with the occurrence of nearly all woodpecker species (Table 2, Fig. 2). GLMMs explained substantial proportion of variation in data (Table 2) and they usually were well parametrized (Figs S1-S8 in Supplementary Information).

**Table 2:** The effect of environmental variables on woodpecker occurrence and species richness in forest patches.

Parametric terms	*Dendropocos major*	*Leiopicus medius*	*Dryobates minor*	*Dryocopus martius*	*Jynx torquilla*	*Picus canus*	*Picus viridis*	*Woodpecker species richness*
Intercept (SE)	1.724 (0.381) ***	− 12.598 (4.412) ***	− 5.935 (0.967) ***	− 4.303 (0.620) ***	− 4.703 (0.803) ***	− 7.356 (1.615) ***	− 4.251 (0.582) ***	− 0.166 (0.064) **
Patch size	1.452 (0.294) ***	2.783 (1.724)	1.191 (0.788)	0.899 (0.353)*	− 0.959 (0.498)*	0.268 (0.696)	0.104 (0.414)	0.244 (0.050) ***
Nearest neighbour distance	− 0.384 (343)	1.234 (1.348)	** − 1.508 (0.789) `**	− 0.048 (0.412)	0.964 (0.629)	1.041 (0.934)	− 0.359 (0.461)	− 0.007 (0.066)
Proximity index	− 0.101 (0.350)	0.007 (1.196)	− 2.444 (0.904) **	− 0.214 (0.412)	0.709 (0.597)	1.064 (1.051)	0.391 (0.492)	0.016 (0.072)
Shape index	− 0.394 (0.193) *	− 1.152 (0.961)	− 0.176 (0.468)	− 0.432 (0.272)	***0.579 (0.301)`***	0.791 (0.582)	0.404 (0.294)	− 0.007 (0.037)
Forest age	0.318 (0.209)	2.324 (1.727)	− 0.161 (0.514)	0.229 (0.322)	0.206 (0.385)	0.200 (0.713)	0.489 (0.330)	0.143 (0.046) **
Percentage of coniferous trees	− 0.267 (200)	0.484 (1.485)	− 0.587 (0.673)	0.241 (0.378)	0.156 (0.334)	1.414 (1.018)	− 1.006 (0.375) **	− 0.120 (0.046) **
Proportion of dominant tree species	− 0.374 (0.179) *	− 0.738 (1.206)	0.608 (0.434)	− 0147 (0.322)	0.120 (0.309)	0.724 (0.573)	0.072 (0.300)	** − 0.081 (0.041)** *
Temperature	0.072 (0.223)	0.111 (0.647)	0.174 (0.538)	− 0.413 (0.288)	− 0.255 (0.375)	0.504 (0.768)	− 0.455 (0.261)	− 0.037 (0.046)
Clouds	− 0.089 (0.165)	− 0.321 (0.483)	− 0.276 (0.419)	− 0.131 (0.207)	0.170 (0.286)	0.761 (0.553)	− 0.275 (0.223)	− 0.043 (0.032)
Date	− 0.089 (0.165)	− 0.781 (0.442)	0.330 (0.507)	0.375 (0.280)	0.808 (0.432)	− 0.494 (0.554)	0.305 (0.246)	− 0.011 (0.036)
Survey duration	0.898 (0.214) ***	0.590 (0.576)	0.205 (0.573)	0.483 (0.275)	0.692 (0.421)	− 0.008 (0.649)	0.193 (0.289)	0.230 (0.041) ***
Number of other woodpecker species	− 0.084 (0.183)	0.872 (0.492)	0.409 (0.367)	0.456 (0.217) *	0.587 (0.290)*	1.237 (0.479)**	0.317 (0.248)	** − **
% of marginal variance explained	46.7	94.9	73.0	66.2	42.0	81.9	31.5	43.5

Results are based on generalized linear mixed models with binomial and Conway-Maxwell Poisson (for species richness) error distributions. Function parameters slopes are given with standard errors (in brackets). Statistically significant effects are emboldened. Explanations: ***P < 0.001, **P < 0.01, *P ≤ 0.05, ‘ − 0.05 < *P* < 0.06.

Models indicated that forest patch size was positively correlated with the probability of occurrence for the great spotted and black woodpeckers (Table 2, Fig. 2). For the great spotted woodpecker, the probability of occurrence increased with the forest patch size up to a point, after which the rate of increase slowed (Fig. 2A). In contrast, the occurrence probability for the black woodpecker increased only in larger forest patches (Fig. 2B). Additionally, forest patch size was negatively correlated with the occurrence of the wryneck (Table 2, Fig. 2C).

GLMM indicated that nearest-neighbour distance was negatively correlated with the lesser spotted woodpecker (Table 2, Fig. 2E). The second isolation metric, the proximity index, was negatively associated with the occurrence of the lesser spotted woodpecker, indicating its preference for more isolated forest patches (Table 2, Fig. 2F).

The shape index significantly influenced the occurrence probability of the great spotted woodpecker and wryneck. Great spotted woodpecker occurrence probability was the highest in more compact forest patches (Table 2, Fig. 2G) while the reverse was found for wryneck (Table 2, Fig. 2H).

### The effects of other forest characteristics on the occurrence of individual species

The proportion of coniferous trees negatively correlated with the occurrence probability of the European green woodpecker (Table 2, Fig. 2J).

The occurrence probability of the great spotted woodpecker decreased as the proportion of dominant tree species increased (Table 2, Fig. 2L).

Among other variables, survey duration was positively associated with the occurrence of the great spotted woodpecker (Table 2).

### Effects of fragmentation metrics and other variables on woodpecker species richness

Woodpecker species richness increased with forest patch size (Table 2, Fig. 2D). It did not respond to other fragmentation metrics. Woodpecker species richness increased with forest age (Table 2, Fig. 2I). The richness was the highest in deciduous forests and decreased with increasing percentage of coniferous species within forest stands (Table 2, Fig. 2K). Moreover, share of dominant tree species negatively correlated with woodpecker species richness (Table 2, Fig. 2M). Survey duration was positively associated with the number of woodpecker species recorded per survey (Table 2).

### Species composition and co-occurrence of woodpecker species

**Fig. 3:**
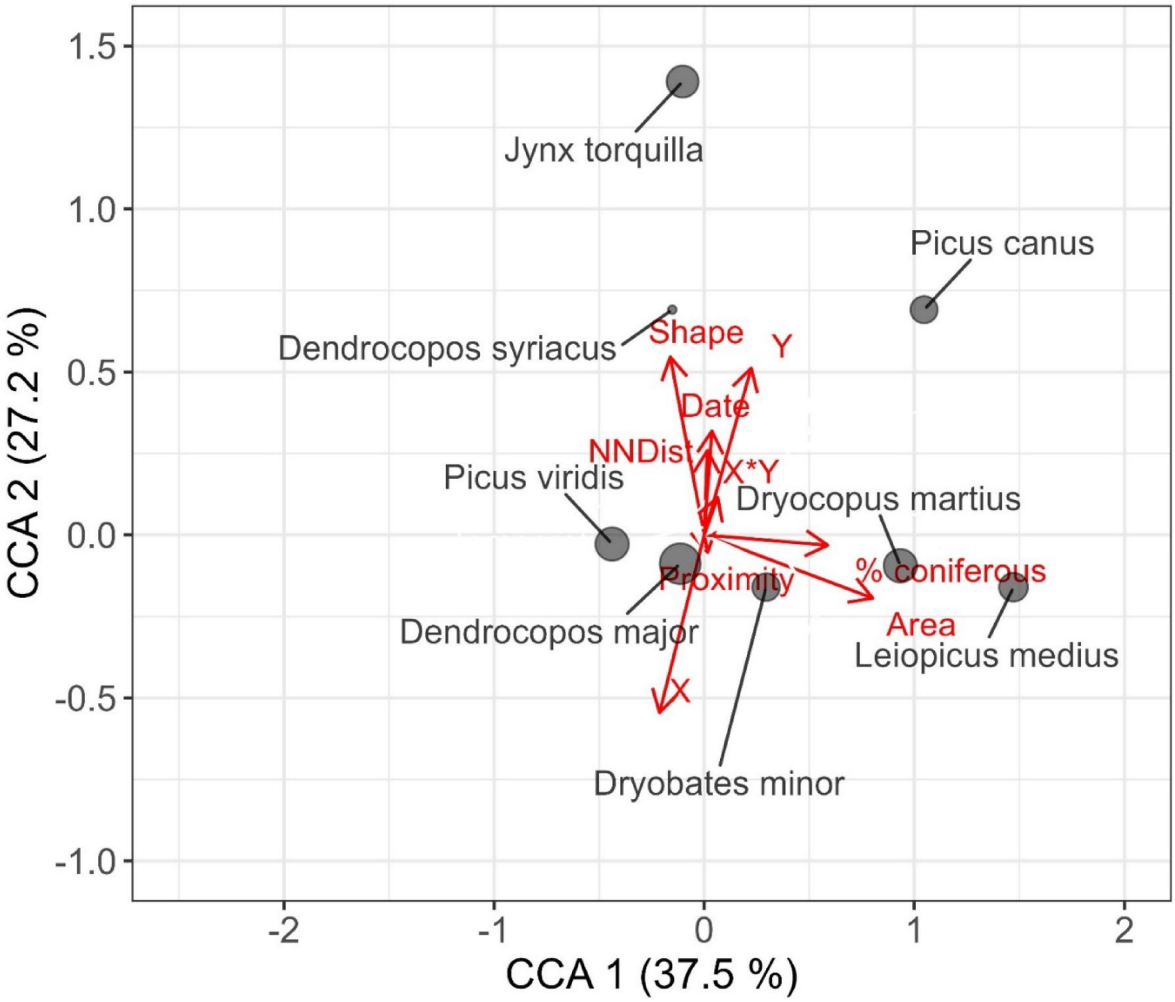
Biplot of factors (in red) influencing woodpecker species composition (in grey) in the studied forest patches.

The results of the canonical correspondence analysis partially confirmed the models for individual species (Table 3). Forest patch size, isolation metrics, and shape index were key variables that differentiates the woodpecker community. Moreover, the proportion of coniferous trees was significant variable in the species ordination (Table 3, Fig. 3). Spatial autocorrelation and survey date also significantly shaped woodpecker species composition, indicating its spatial predictability as well as within-season variation.

**Table 3:** The ordination of woodpecker species and environmental variables in forest patches.

Effect	Df	F	*P*
log(Area)	1	16.113	**0.001**
log(NNDist)	1	2.824	**0.006**
log(Proximity_index + 0.01)	1	4.196	**0.002**
log(SI)	1	4.849	**0.001**
Age	1	0.818	0.551
log(Perc_coniferous + 1)	1	3.594	**0.001**
Share_dominant	1	1.667	0.130
Temperature	1	1.405	0.212
Date	1	3.044	**0.001**
log(Survey_duration)	1	1.938	0.059
X_coord	1	2.192	**0.045**
Y_coord	1	3.966	**0.002**
X_coord*Y_coord	1	4.073	**0.002**
Residual	333		

Results from canonical correspondence analysis. *Df* effective degrees of freedom, *F* F-statistics. Statistically significant effects are emboldened.

GLMMs for three woodpecker species showed that their occurrence was positively correlated with the species richness of other woodpeckers (Table 2, Fig. 2N–P).

### Woodpeckers and other bird species

**Fig. 4:**
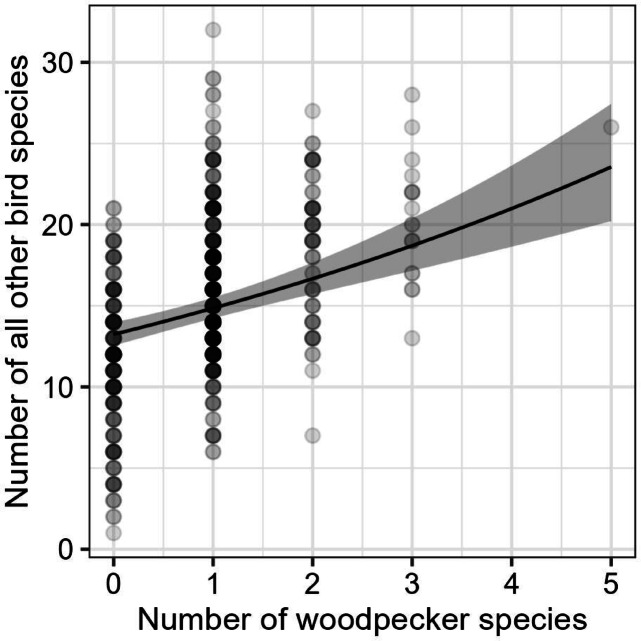
The association between number of woodpecker species and the species richness of all other birds per survey in the studied forest patches, based on results from the generalised linear mixed model.

GLMM (slope: 0.115 ± 0.018, variance explained = 9%) showed that number of woodpecker species was positively correlated with number of other bird species after controlling for forest identity and spatial autocorrelation (Fig. 4).

## Discussion

Our study reveals that woodland patch size, isolation and shape affect the occurrence and species richness of woodpeckers. However, individual species respond differently, and other patch characteristics, such as the percentage of coniferous tree species in woodland patches, proportion of dominant tree species in main forest canopy, and the age of the forest stand also play a significant role in shaping the woodpecker community.

Our results partially confirmed our first hypothesis, which predicted that woodpecker species occurrence would increase with forest patch size and decrease with spatial isolation. This was true for several woodpecker species, such as great spotted woodpecker, black woodpecker and overall woodpecker species richness. Our study supports previous findings that patch size as a critical variable in determining habitat suitability for a given species or entire communities^13,38–40^. However, the wryneck, a secondary cavity-nester among European woodpeckers, was more frequently found in smaller tree stands, likely due to its association with open landscapes^41^.

Other fragmentation metrics had less frequent significant effects on woodpecker species occurrence, and the these effect varied between species. This suggests that for the woodpeckers, the presence of large intact forest patches may be more important than the spatial configuration of those patches within the landscape. The insignificance of nearest-neighbour distance in shaping the occurrence of most woodpecker species indicates that isolation plays a minimal role for woodpeckers except perhaps those small-bodied species. Some studies have suggested that the amount of suitable habitat within a landscape is one of the key factors influencing the diversity of saproxylic beetles, which constitute a large proportion of the woodpecker diet^42^. Our results partially support these findings, with forest patch size being a significant factor in shaping the occurrence of woodpeckers, while the proximity index was notably only significant for the lesser spotted woodpecker.. Moreover, shape index was negatively associated with the occupancy of the most common species – great spotted woodpecker, but positively the occupancy of the wryneck. This indicates that edge effect, not necessarily associated with patch size, may have contrasting effects on woodpecker species.

Wryneck, along with lesser spotted woodpecker and European green woodpecker, is mostly associated with open landscapes or ecotonal habitats^41^. Moreover, European green woodpecker feeds on ants and inhabits these areas more frequently than the interiors of large forests^41,43–46^. This may explain the difference in habitat preferences between these species and the other woodpecker species, which are typically more abundant in forest habitats^7,22,25^. Data for the Syrian woodpecker were too sparse to confirm similar patterns for this species.

Positive association between patch size and woodpecker species richness may be explained by fact that woodpeckers, being territorial species, require a minimum area to establish territories, which they often occupy year-round^40,47,48^. Thus, large woodland patches may provide more suitable niches for different woodpecker species. At the same time woodpeckers may be affected by metapopulation dynamics, with smaller woodland patches experiencing more frequent local extinctions/emigrations, resulting in fewer individuals and species compared to larger woodland patches^47,49^.

Our results only partially confirmed our hypothesis that woodpecker species occurrence probability would increase with forest patch age and decrease with the homogenisation of tree composition within forest patches. None species occurrence was associated with forest age but woodpecker species richness was. This partially contradicts other studies from Europe, which found that a number of individual woodpecker species (like other woodland bird species) prefer older forest patches^18,19,48,50^. This preference is linked to the availability of food resources and suitable nesting sites or substrate^51,52^. Our study confirmed, however, that woodpeckers probably prefer heterogenous forests containing a proportion of deciduous species^18,53,54^. One key explanation for this preference may be the year-round availability of food sources. Previous studies have indicated saproxylic and ground-dwelling beetles (which form a significant part of the woodpecker diet) diversity increases with forest age^55–57^. Older forest stands are also characterised by higher deadwood abundance, providing preferred habitats for many saproxylic species^58^ and some ant species, such as *Formica rufa*^46^. Ants are the primary food source for some woodpecker species like the black woodpecker and European green woodpecker^44^. For example, the European green woodpecker feeds in older stands during the winter but shifts to feeding on different ant species in young forest plantations during the summer^46^.

Our study confirmed woodpeckers’ preference for heterogenous forests containing a proportion of deciduous species^18,53,54^. The abundance of saproxylic beetles and ants may also be an important factor in explaining the decreasing woodpecker occupancy and woodpecker species richness with increasing forest stand homogenisation, measured by the increase of the percentage of coniferous species or the dominance of a single tree species in main forest canopy. Other studies have found that increased tree species diversity leads to higher deadwood abundance^59^ and invertebrate species richness^60,61^.

Despite the different response of some woodpecker species to variables associated with habitat fragmentation and quality the positive association between occurrences of some species was evident. Notably, the presence of one woodpecker species may positively influences the detection of others, especially among more specialised species such as the middle spotted woodpecker, lesser spotted woodpecker, and grey-headed woodpecker. Moreover, our results confirm previous research that woodpecker can serves as good indicators of species richness of other bird species^54^. The positive associations among woodpecker species occurrences may suggests low competition for resources, with communities forming based on individual species’ responses to forest characteristics. It may be especially true in large woodland patches that provide high availability of various resources. For example, the middle spotted woodpecker prefers dead wood branches in large, living trees for excavation^44,52^, while the black woodpecker favours thick trunks with no branches^40,48^. In contrast, the great spotted woodpecker is most opportunistic species in its choice of excavation sites^40^.

## Study limitations

Our study has certain limitations that should be acknowledged when interpreting the results in broader ecological contexts. First, the bird counts were conducted during a single year and exclusively during the breeding season. Woodpecker occurrence may vary across years due to interannual variability and factors unrelated to breeding activity. Second, we did not account for imperfect detectability. However, we included several variables associated with detectability, such as survey weather conditions, to mitigate this potential bias. Third, although experienced observers conducted the surveys, differences in detection abilities between observers may have introduced observer bias. When we modelled woodpecker species richness, we were unable to fully account for underdispersion in residuals. However, after testing multiple modelling approaches, we consistently obtained similar results, indicating that the observed associations are likely genuine. Finally, while our study focused heavily on fragmentation metrics, other factors—such as microhabitat structure, particularly the availability of deadwood, and human disturbance, were not considered, despite their known importance for woodpecker species.

## Electronic supplementary material

Below is the link to the electronic supplementary material.

Supplementary Material 1.

## Data Availability

All data are attached to the manuscript and will be made available upon request to authors.
