# Supplementary material for: Forest fragmentation and heterogeneity shape the occurrence of woodpecker species in Central Europe
Source: Sci Rep. 2025 Jul 1;15:21660. doi: 10.1038/s41598-025-04832-5 (PMC12216957; doi:10.1038/s41598-025-04832-5)
Supplement: Supplementary file 1 — Supplementary Material 1. [file 41598_2025_4832_MOESM1_ESM.docx]

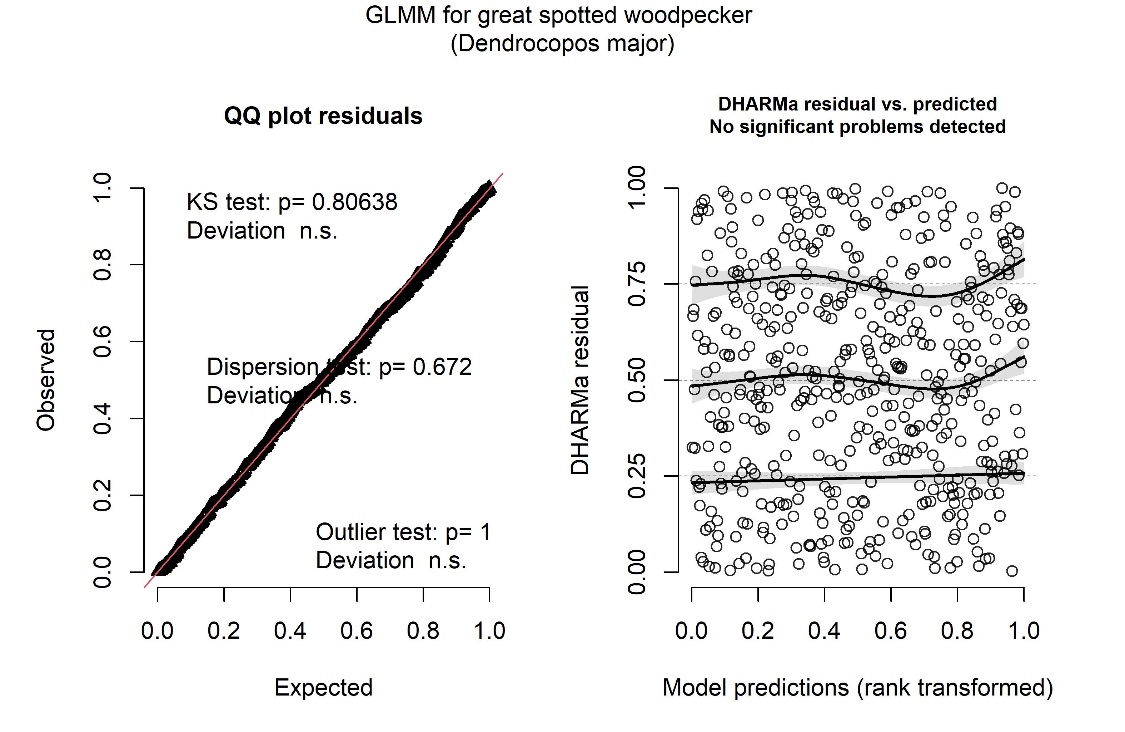


Fig. S1. Diagnostics for the GLMM explaining the occurrence of the great spotted woodpecker in forest patches.


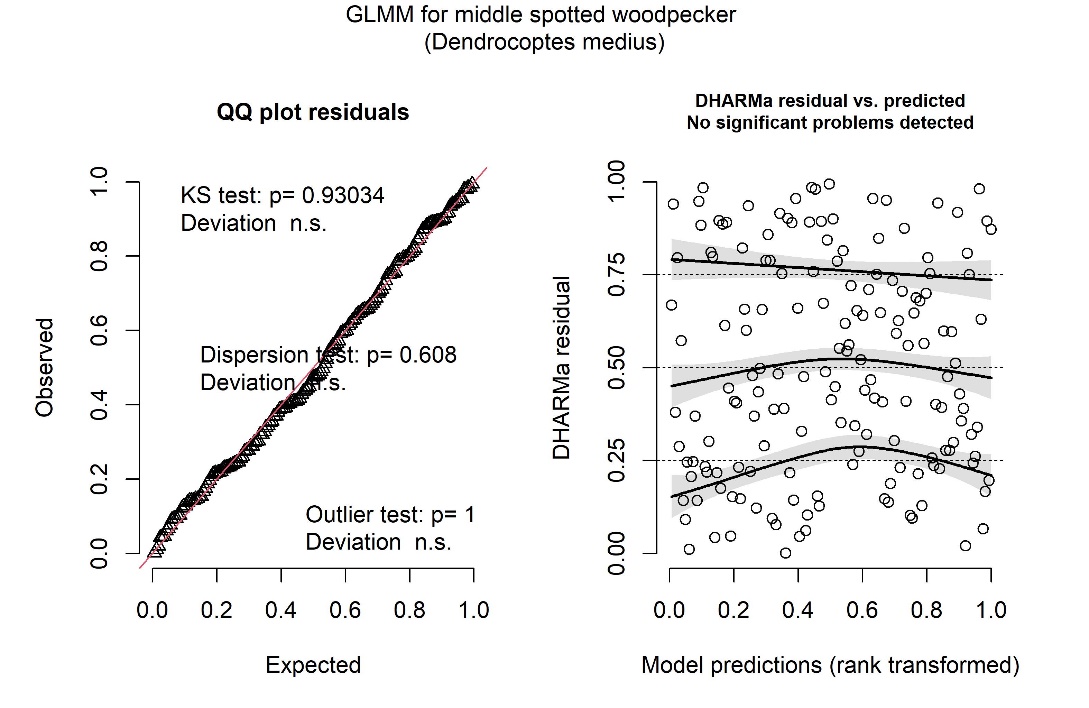


Fig. S2. Diagnostics for the GLMM explaining the occurrence of the middle spotted woodpecker in forest patches


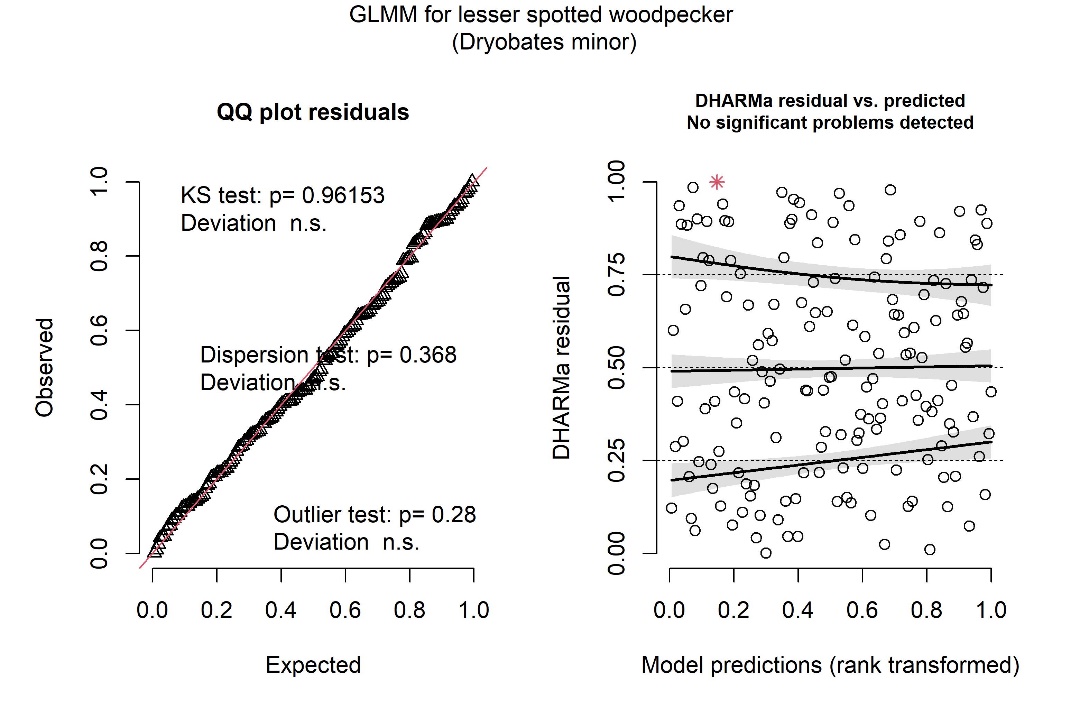


Fig. S3. Diagnostics for the GLMM explaining the occurrence of the lesser spotted woodpecker in forest patches


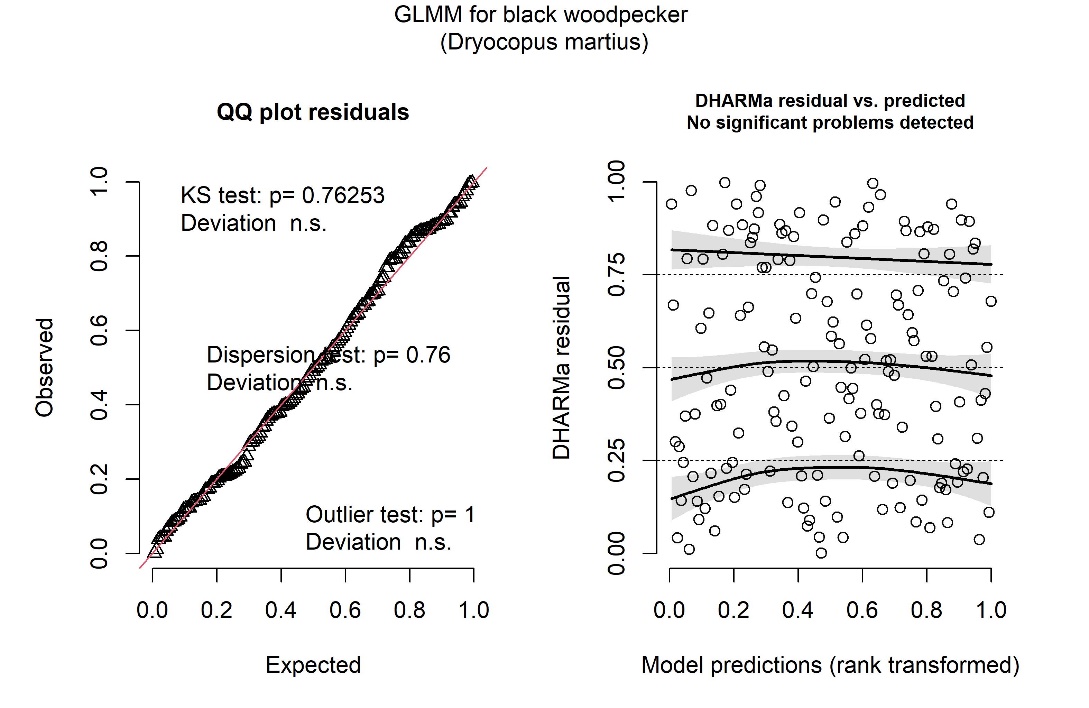


Fig. S4. Diagnostics for the GLMM explaining the occurrence of the black woodpecker in forest patches.


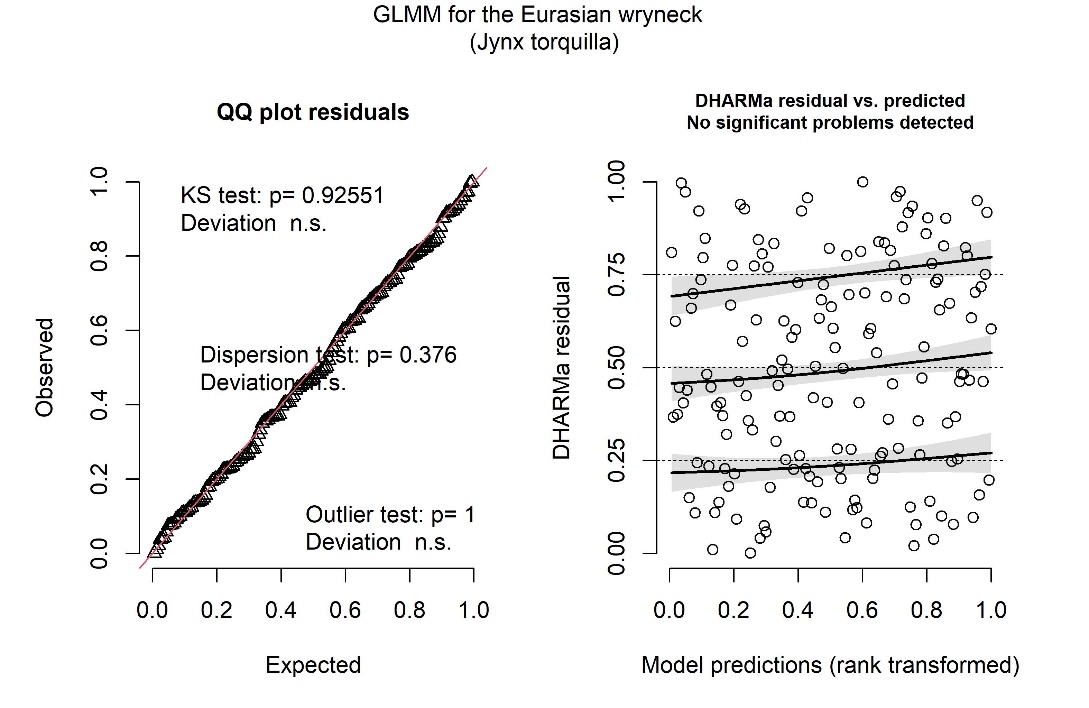


Fig. S5. Diagnostics for the GLMM explaining the occurrence of the Eurasian wryneck in forest patches.


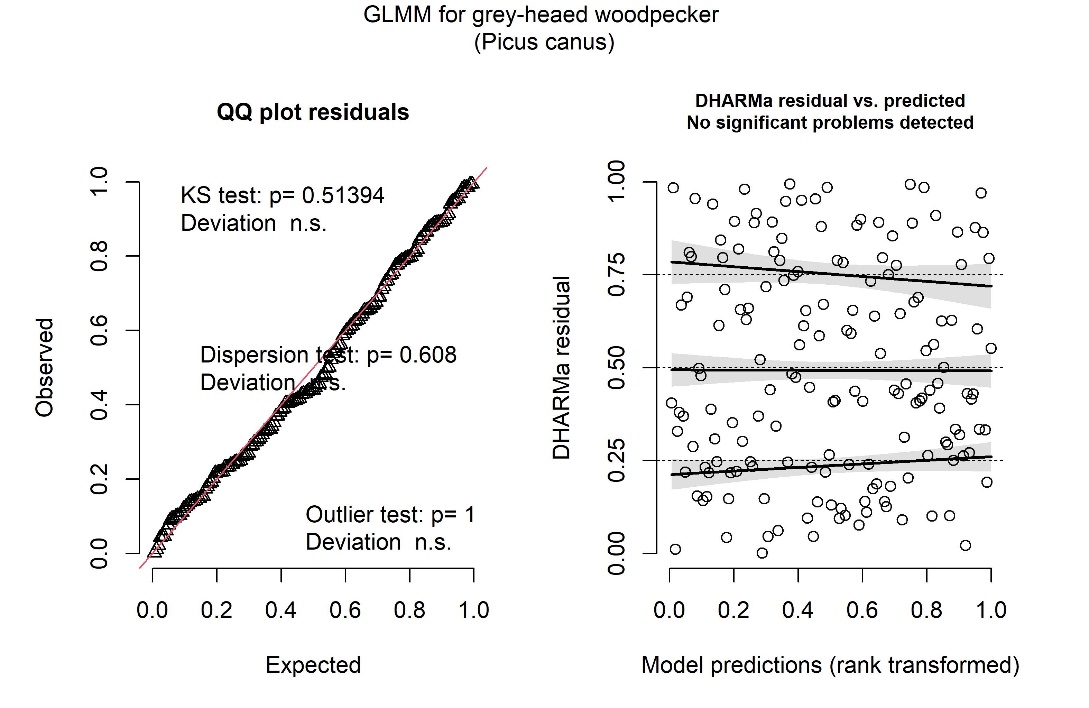


Fig. S6. Diagnostics for the GLMM explaining the occurrence of the grey-headed woodpecker in forest patches.


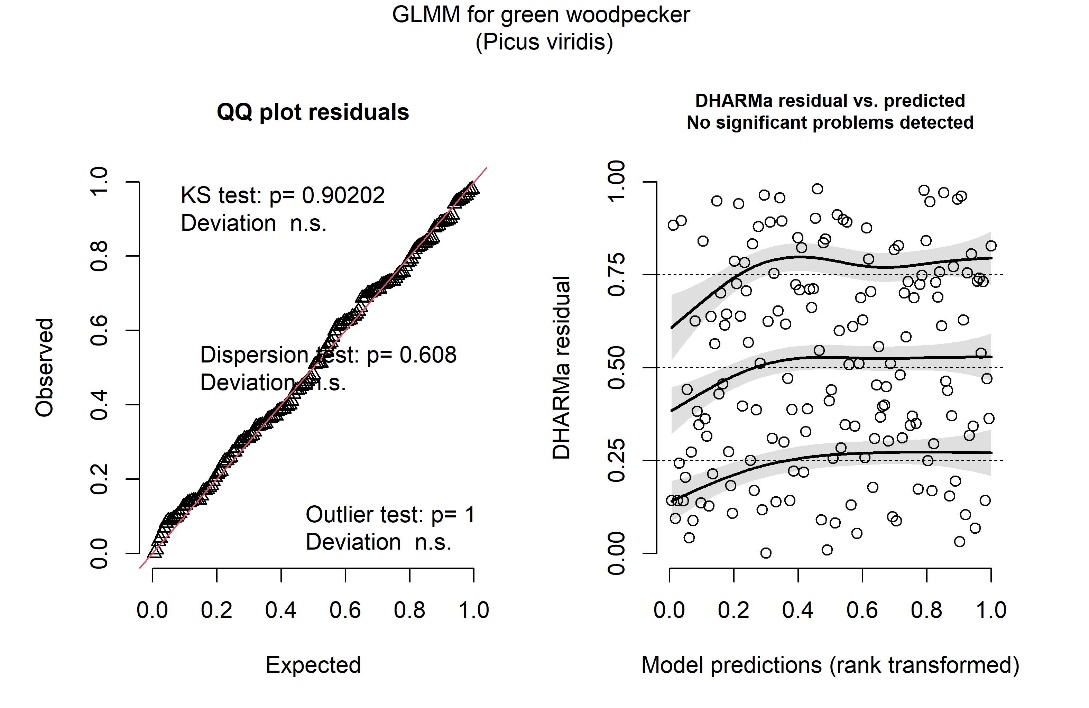


Fig. S7. Diagnostics for the GLMM explaining the occurrence of the green woodpecker in forest patches.


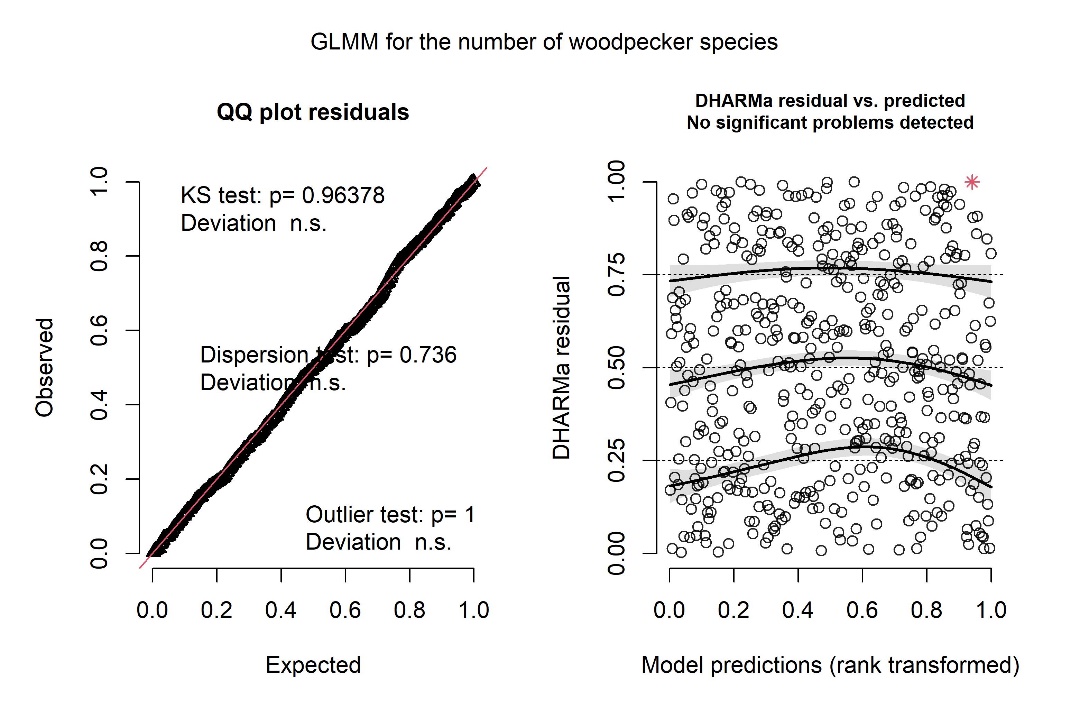


Fig. S8. Diagnostics for the GLMM explaining the number of woodpecker species in forest patches.
